# Evaluation of Perfusion Index as a Screening Tool for Developing Critical Limb Ischemia

**DOI:** 10.3400/avd.oa.21-00100

**Published:** 2021-12-25

**Authors:** Nobuko Yamamoto, Hideki Sakashita, Noriyuki Miyama, Kanako Takai, Hiroyoshi Komai

**Affiliations:** 1 Department of Vascular Surgery, Kansai Medical University Medical Center, Moriguchi, Osaka, Japan

**Keywords:** peripheral artery disease, critical limb ischemia, perfusion index

## Abstract

**Objective**: The perfusion index (PI) is a physiological marker for evaluating the peripheral circulation. We explored the possibility of using PI as a screening tool for development of critical limb ischemia in peripheral artery disease (PAD).

**Method**: We measured the PI in 79 limbs of 70 PAD patients. Data were analyzed to find a correlation between the PI and PAD severity.

**Result**: The PI tended to be lower as PAD became severer. Especially, there were significant differences between the Fontaine 1 and Fontaine 4 groups in average PI and minimum PI, and between Fontaine 1 and two other groups (Fontaine 2 and Fontaine 4 groups) in maximum PI. A mild correlation was found between PI and the ankle brachial index. These data were used to calculate an average PI of 0.27 as a cut-off value for critical limb ischemia (CLI). In 65 asymptomatic PAD patients and claudication, significantly more patients with a PI value greater than the cut-off value developed CLI than those with a PI lower than the cut-off.

**Conclusion**: The PI can be a useful tool for evaluating the development of CLI in mild PAD patients, and patients tended not to progress to CLI when their average PI was higher than 0.27. (This is a translation of Jpn J Vasc Surg 2020; 29: 103–108.)

## Introduction

Functioning limb salvage is the most important issue in the treatment of critical limb ischemia (CLI) in patients with peripheral arterial disease (PAD). Despite recent advances in revascularization techniques, there are still many cases resulted in lower-limb amputation, thus, early diagnosis of critical ischemia is essential. However, not a few patients develop CLI without an initial symptom of claudication, and we sometimes even lose the chance to perform revascularization. Although prophylactic revascularization before developing CLI is thought to be ideal, the risk of invasive procedures is often beyond the acceptable range as considering the nutritional status and comorbidities of patients with PAD. We have been investigating the difference between patients with intermittent claudication and those with CLI to identify patients with the potential of progression to CLI using biomarkers and vascular endothelial function test^1–7)^; however, we are yet to find a useful marker.

Perfusion index (PI) is a physiological marker representing the ratio of pulsatile to non-pulsatile blood volume in peripheral tissues. It is easily measured by detecting arterial oxygen saturation waveforms by pulse oximeter. PI is an index that can continuously and non-invasively evaluate peripheral perfusion without the need for maintaining constant body positions or for body movements. Recently, it has been used for evaluating the depth of the general anesthesia^8)^ and for monitoring circulatory status in the intensive care of neonates.^9)^ Peripheral circulation can be evaluated in a simple and non-invasive manner, thus, we hypothesized that measuring toe PI can be used to determine the severity of ischemia in patients with PAD.

## Patients and Methods

Seventy-nine limbs of 70 patients (56 limbs in males, 23 limbs in females) with PAD (ankle-brachial index [ABI]<0.9) who presented to our department between March 2015 and August 2016 were included in the study. The patients were put in the supine position for a few minutes at room temperature (approximately 20°C to 25°C) and measured the toe percutaneous oxygen saturation at the every toes on both sides by attaching the oximeter. The device specialized to calculate PI (Radical 7 pulse Co-oximeter (Masimo Corporation, Irvine, CA, USA)) were used to determine PI. The accurate measurements were confirmed by continuous and stable value of the oxygen saturation. For toes in which the PI was non-measurable and for toes without pulsatile flow, the PI value was given 0. PI values were assessed with the patient information and clinical data obtained from their chart. This study was reviewed and approved by the Institutional Review Board of Kansai Medical University Medical Center (Approval No. T28-19), and written consent to participate in the study was obtained from every patient after provided with an information regarding the study.

### Assessment 1

The limbs were classified into 3 groups based on clinical severity according to the Fontaine classification at the time of PI measurement, with 19 limbs classified as F1 (asymptomatic or cold), 46 limbs as F2 (claudication), and 14 limbs as F4 (ulceration and necrosis), and the mean, maximum, and minimum PI of all the toes in the affected limbs were compared for each group. In addition, PI values were compared with the values obtained from conventional blood flow tests, such as ABI and skin perfusion pressure (SPP).

### Assessment 2

Of the aforementioned target patients’ limbs, the 65 limbs in the F1 and F2 groups were grouped together as the non-CLI group, whereas the 14 limbs in the F4 group were defined as the CLI group and the cutoff value for the diagnosis of CLI at the mean, maximum, and minimum PI values was calculated from receiver operating characteristic (ROC) curves.

### Assessment 3

In the F2 group, 6 limbs that developed CLI during the subsequent 1 year or that had SPP of ≤40 mmHg at the initial examination were considered to have the potential severe ischemia (the severe IC group), and the remaining 40 limbs were defined as the mild IC group, and differences in the PI cutoff values between the groups were evaluated.

### Statistical methods

The analysis software used was JMP13.0.0 (13.0). For the analysis of assessment 1, multiple comparisons were performed using the Tukey–Kramer honest significant difference test for the mean, maximum, and minimum PI values in each of the F1, F2, and F4 groups. Additionally, the correlation coefficient and p values were calculated using a bivariate normal ellipse for correlations with ABI and SPP. For the analysis of assessment 2, cutoff values, area under the curve (AUC), and p values were determined from ROC curves using logistic regression. For the analysis of assessment 3, contingency tables were generated using cutoff values determined in assessment 2, and p values were calculated using chi-squared tests.

A p value <0.05 was considered statistically significant.

## Results

### Patient background characteristics

There were no significant differences in age; presence of diabetes, hypertension, and dyslipidemia; smoking history; and insulin use among the F1, F2, and F4 groups. There were 8 hemodialysis patients in the F4 group (57%), which was significantly higher than those in the other 2 groups (**Table**).

**Table 1:** Table Baseline demographic characteristics

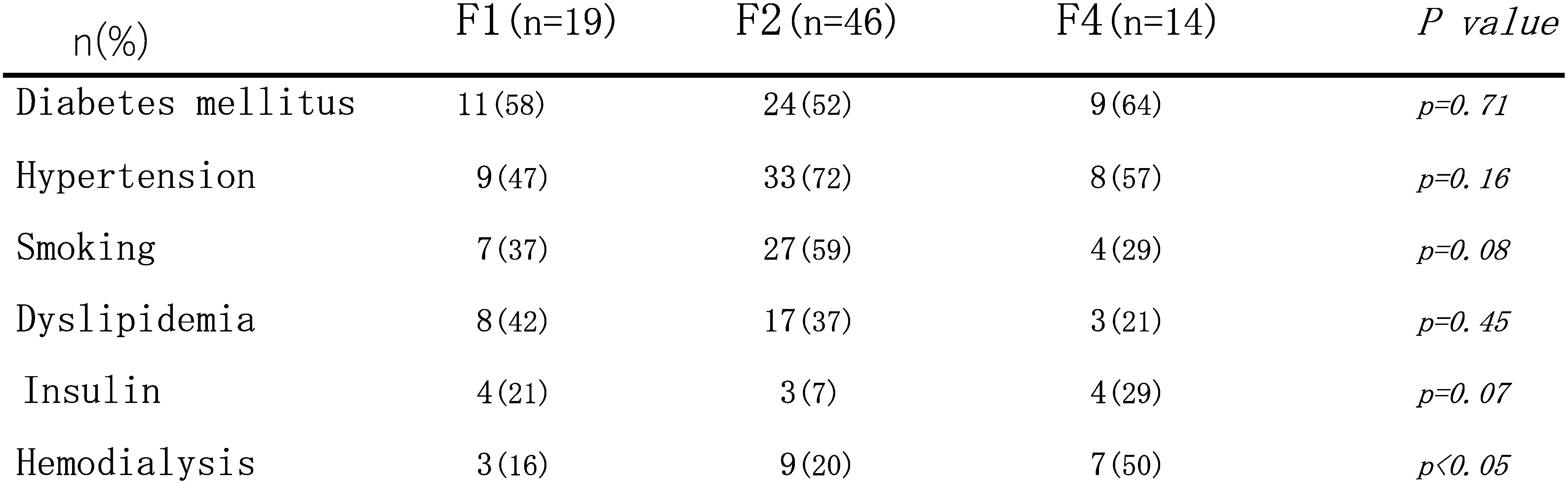

### Analysis 1

**Figure 1:**
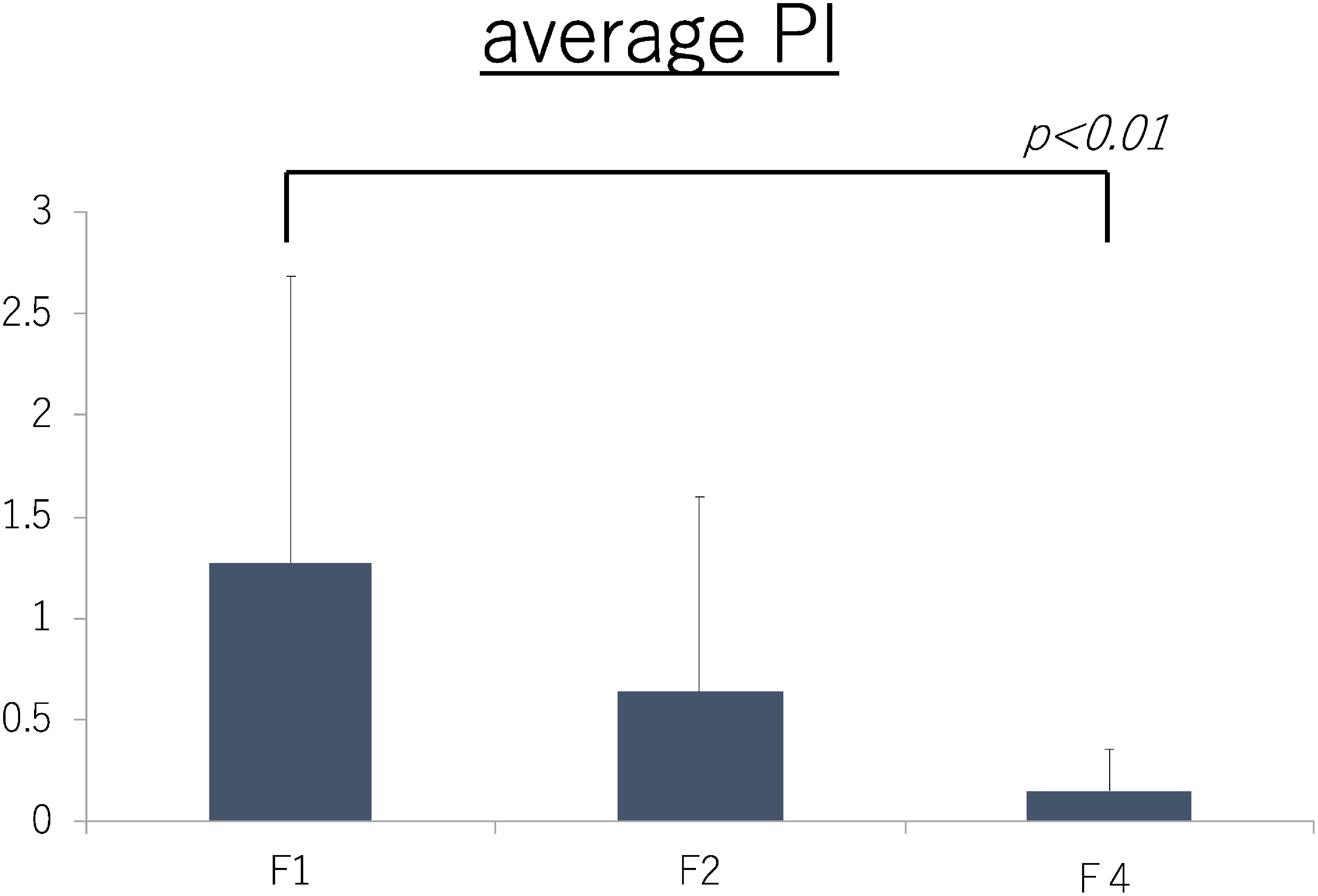
Fig. 1 Univariate correlation of PI average value. PI average value in F1 group is significantly higher than that in F4 group.

The mean PI was 1.27±0.23 in the F1 group, 0.64±0.15 in the F2 group, and 0.15±0.27 in the F4 group, showing significantly higher values in the F1 group than in the F4 group (F1 vs. F4 group, p<0.01) (**Fig. 1**).

**Figure 2:**
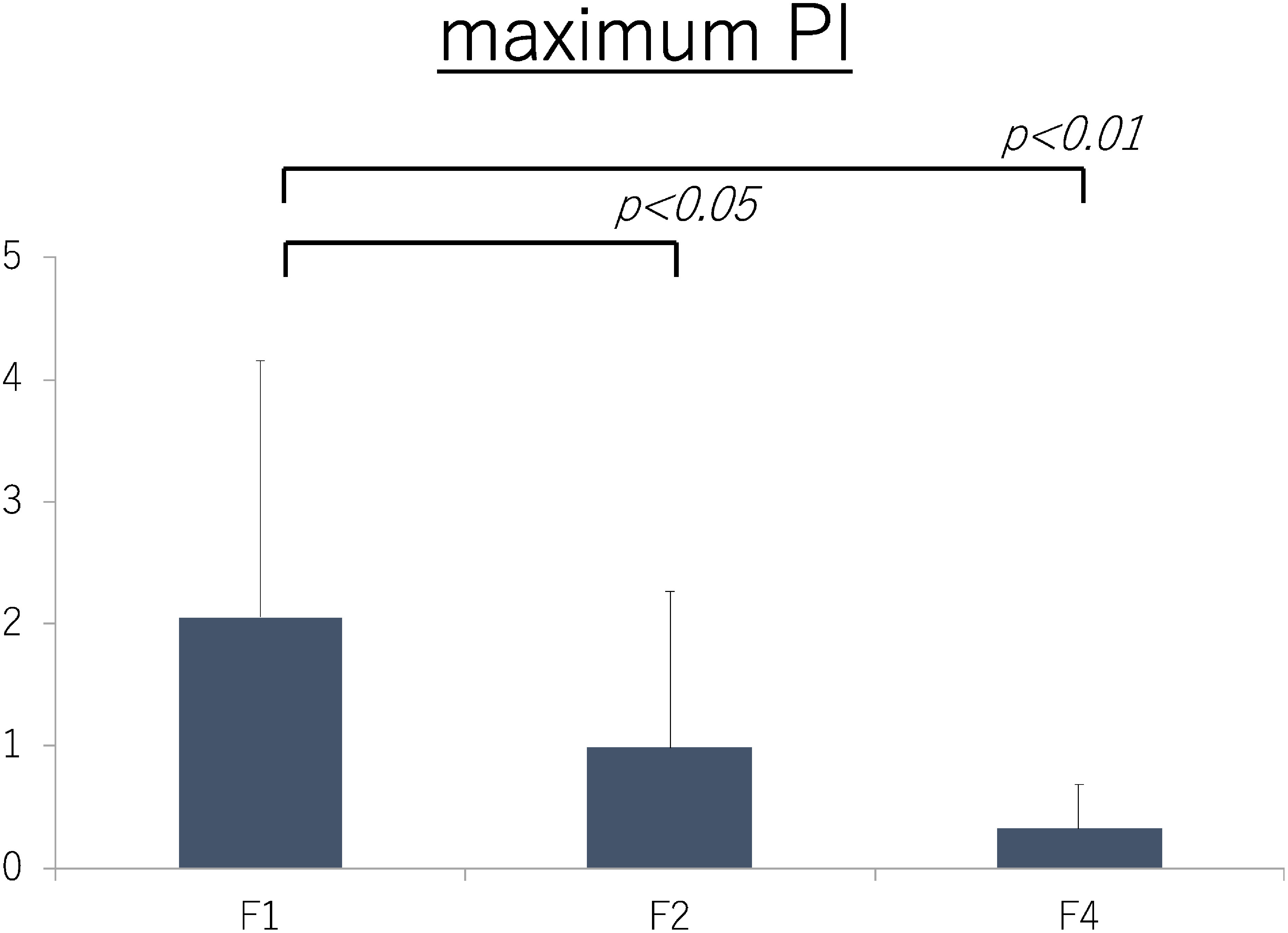
Fig. 2 Univariate correlation of PI maximum value. PI maximum value in F1 group is significantly higher than those in other two groups, but there is no significant difference between F2 and F4 group.

The maximum PI was 2.06±0.33 in the F1 group, 1.00±0.21 in the F2 group, and 0.32±0.38 in the F4 group, showing significantly higher values in the F1 group than in the F2 and F4 groups (F1 vs. F2 group, p<0.05; F1 vs. F4 group, p<0.01) (**Fig. 2**).

**Figure 3:**
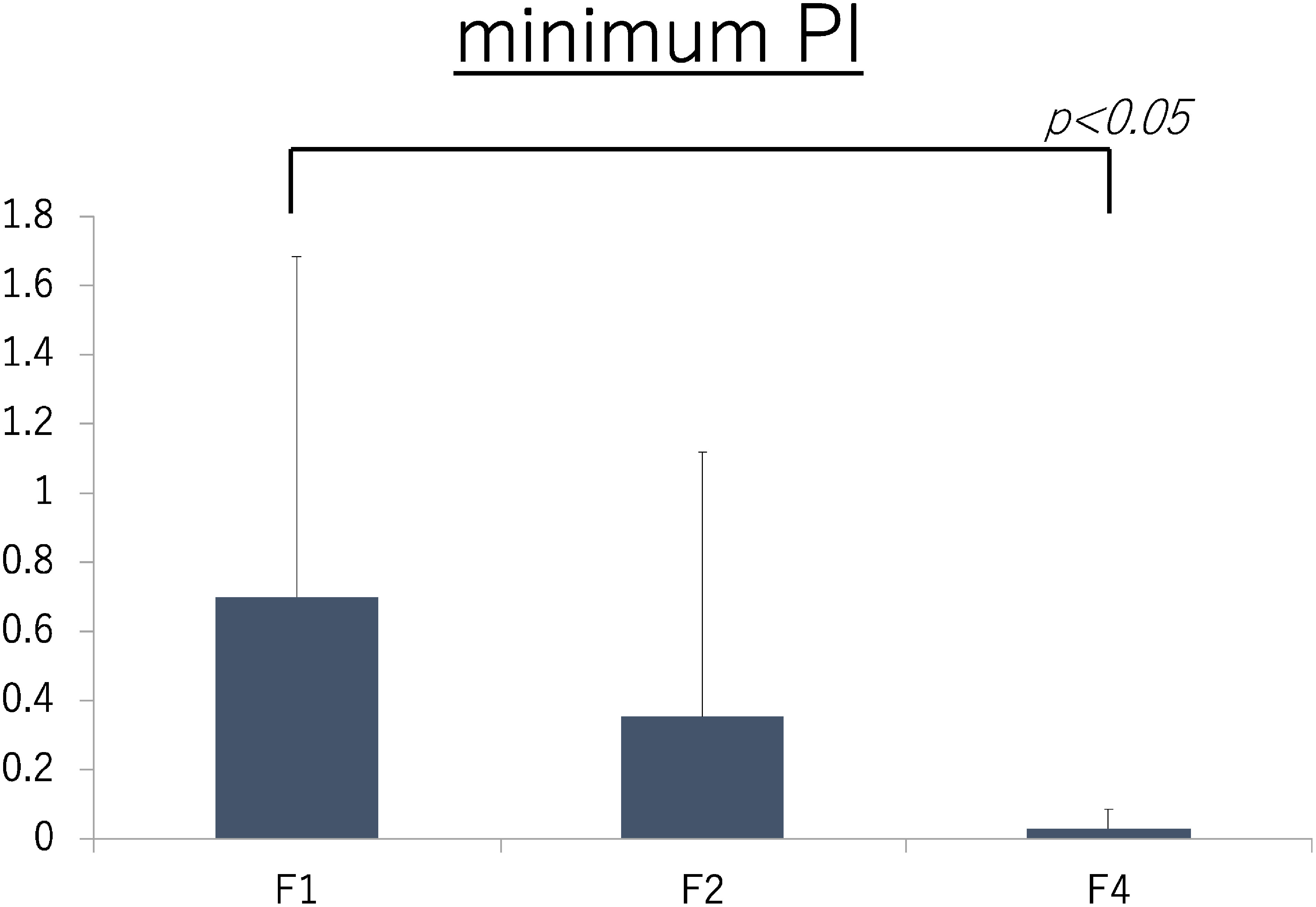
Fig. 3 Univariate correlation of PI minimum value. PI minimum value in F1 group is significantly higher than that in F4 group.

The minimum PI was 0.70±0.17 in the F1 group, 0.35±0.11 in the F2 group, and 0.03±0.20 in the F4 group, showing significantly higher values in the F1 group than in the F4 group (F1 vs. F4, p<0.05) (**Fig. 3**).

**Figure 4:**
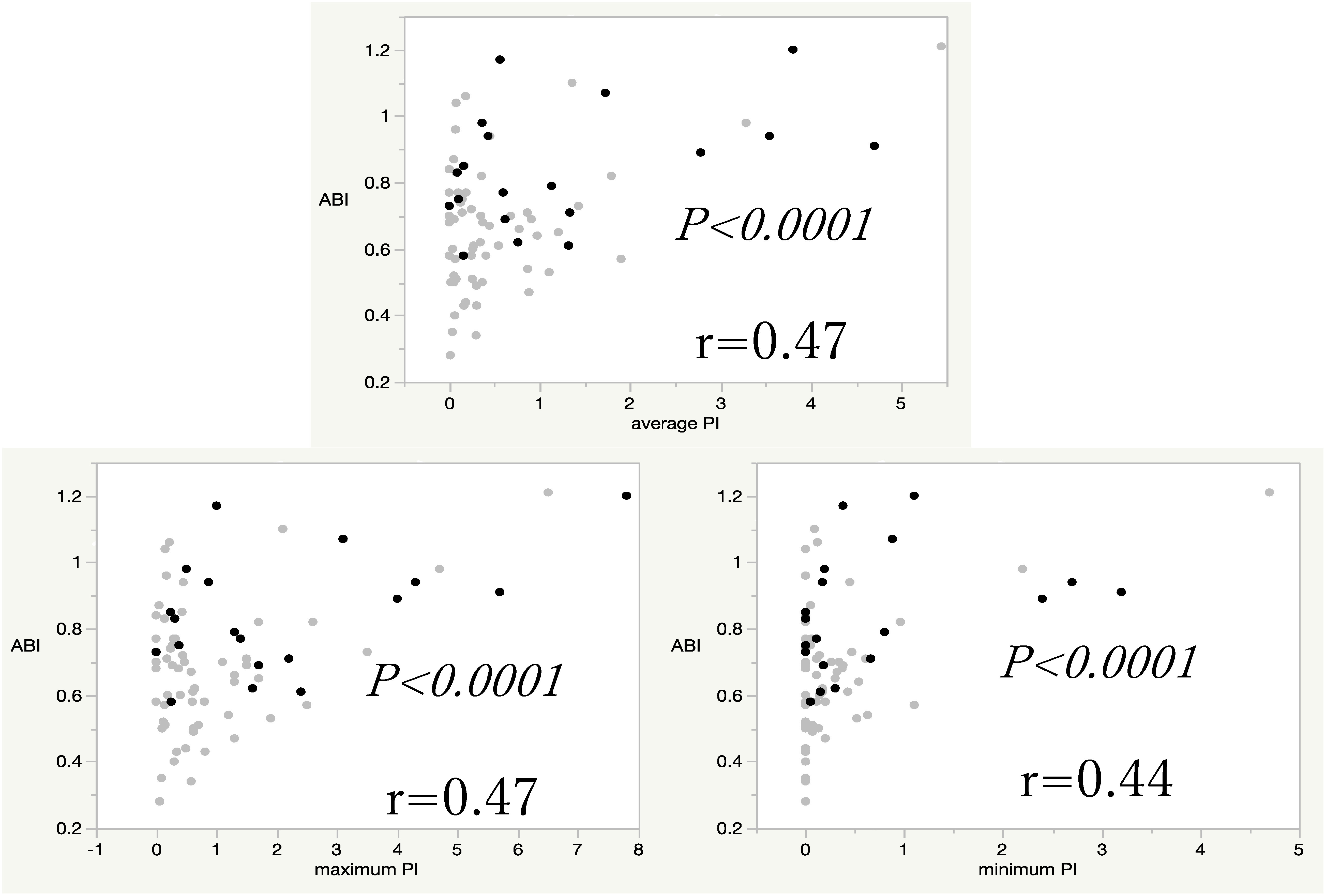
Fig. 4 Correlation analysis between ABI and each PI value. There are moderate correlations between ABI and each PI value in all groups.

**Figure 5:**
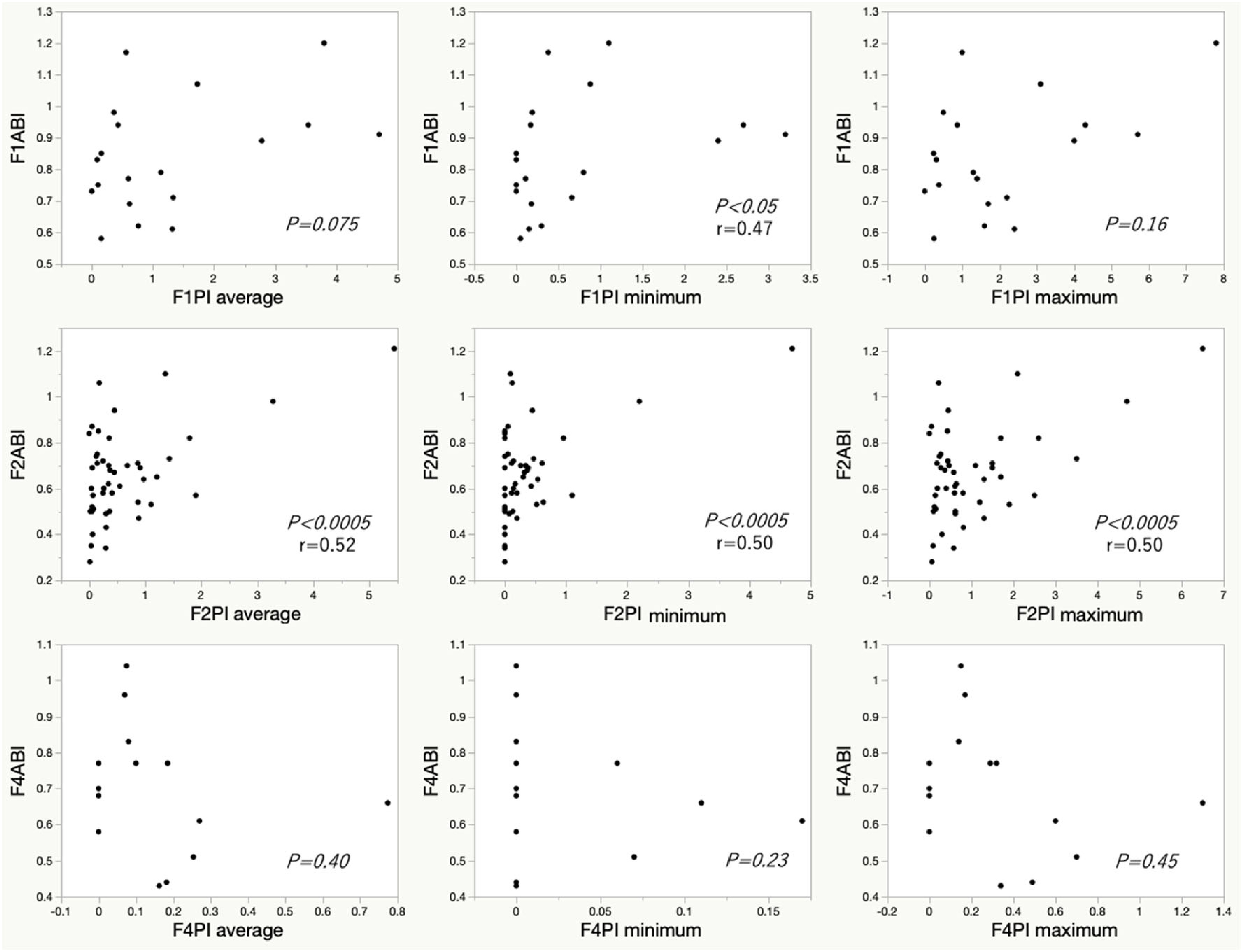
Fig. 5 Correlation analysis between ABI and each PI value in each group. There are moderate correlations between ABI and PI maximum value in F1 group and all PI value in F2 group.

When the correlation with ABI was evaluated in 79 patients for whom measurements could be obtained, a moderate correlation was found with the mean, maximum, and minimum PI (**Fig. 4**). Among these, a significant correlation of all the indices with ABI was observed in the F2 group; however, there were no significant correlations of the indices with ABI in the F1 and F4 groups, except for a weak correlation at the maximum PI in the F1 group (**Fig. 5**).

The SPP was measured simultaneously in 19 patients: 3 in the F1 group (16%), 7 in the F2 group (15%), and 9 in the F4 group (64%). There were no significant correlations between the mean SPP and the mean, maximum, and minimum PI on the affected side in these patients.

### Analysis 2

When the PI cutoff values for CLI at PAD were calculated from the ROC curves, the cutoff was found to be 0.27 (AUC=0.76648, p<0.01) at the mean PI, 0.34 (AUC=0.75275, p<0.01) at the maximum PI, and 0.11 at the minimum PI (AUC=0.77253, p<0.01).

### Analysis 3

**Figure 6:**
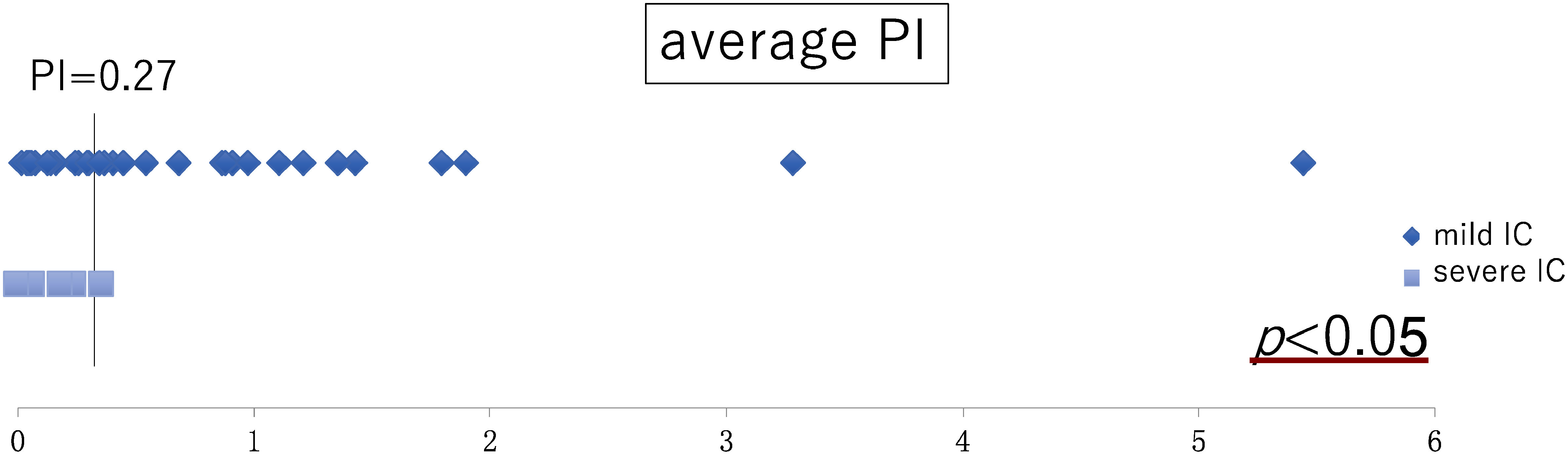
Fig. 6 Cut off value of PI average in CLI is set on 0.27. Larger number of PI average in severe IC group are lower than cut off value.

Among the cutoff values obtained in analysis 2, the mean PI value, which was considered to cover the variation among the toes, was applied to the mild IC group and the severe IC group to see if it could classify the severity of the patients. The number of patients at or below the cutoff value was found to be significantly higher in the severe IC group than those in the mild IC group (p<0.05) (**Fig. 6**). The sensitivity, specificity, positive predictive value, and negative predictive value for determining severe IC with this cutoff were 83.3%, 62.5%, 25%, and 96%, respectively; thus, if the value was at or above the cutoff, then the likelihood of developing severe IC was considered to be low.

## Discussion

Recently, the number of vascular procedure, especially endovascular interventions, for PAD patients has been steadily increasing, whereas the number of major amputation has been declined.^10)^ It implicates that it might be important to undergo effective revascularization at the appropriate timing. Intermittent claudication is present as a preliminary symptom in only 35% of patients with CLI.^11)^ Moreover, half of the patients who required lower-limb amputation were reportedly asymptomatic 6 months before.^12)^ While conservative therapy based on drug and exercise is recommended for claudication, once a CLI with ulcer/necrosis occurs, it takes a lot of time and effort to complete wound healing, even if revascularization is performed at an early stage.^13)^ Assumably it can be a heavy burden on the medical economy. Thus, this study aimed to identify the possibility of developing CLI in the future among patients with mild PAD. In other words, a precise diagnosis or prediction of the transition point from claudication (or asymptomatic ischemia) to critical ischemia would allow for timely revascularization, thereby achieving limb salvage at minimal effort.

Unlike conventional pulse oximeters, the Radical 7 developed by the Masimo Corporation was capable of measuring oxygen saturation only in arterial blood using original signal extraction technology (SET), thereby leading to successful, accurate oxygen saturation measurements with less noise. With this SET, all optical density ratios corresponding to the oxygen saturation of 1% to 100% are analyzed, and the maximum peak is calculated as the arterial oxygen saturation.^14)^ PI is obtained by calculating the ratio of pulsatile to non-pulsatile blood volume obtained by analyzing the arterial oxygen saturation waveforms. A high PI indicates a high pulsatile blood volume and more arterial blood, thereby indicating good peripheral circulation. The advantages of measuring PI include the ability to quantify the amount of arterial blood reaching each toe; the ability to perform repeated measurements easily and in a short time, even at outpatient clinic; and the ability to perform measurements in a non-invasive manner with minimal pain. Compared with conventional blood flow test, such as ABI and SPP, PI can be easily measured as objective data in daily medical practice without visiting a laboratory. Thus, bedside measurements can be performed during invasive treatments and in patients who have difficulty in moving. Although PI is a handy tool, there are some points to be checked for accurate measurement. As PI values may be affected with sympathetic nerve condition,^8)^ it is important to not expose the patient to cold stimulation or strong tension at the time of measurement. In addition, care should be taken to ensure that the detector is applied to the nail in perpendicular to the toe as there is a possibility that the position of the probe may be altered by the examiner. In the preliminary experiment, the intra-rater reliability for the intraclass correlation coefficient was found to be 0.85 for 10 measurements, and the inter-rater reliability was found to be 0.87 for 5 raters, both acceptable values.

In analysis 1, significant differences were found between the F1 and F4 groups for all the indices, and as a whole, the PI value decreased with increasing severity of PAD. The absence of significance between the F2 group and the other groups may be due to the variable severity of intermittent claudication in the F2 group. On the other hand, the correlation with ABI was strongest in the F2 group, whereas the correlation was weak in the F4 group. This may be partly because ABI was not accurately measured due to calcification, particularly Monckeberg’s calcification in the patients with hemodialysis, many of whom in the F4 group.^15)^ Although SPP is now widely used for assessing the severity of PAD, particularly in CLI,^16)^ no correlation with PI was observed in the present analysis. The main reasons for this were that the number of patients in whom both SPP and PI were measured was small, and that most of the patients in whom SPP was measured belonged to the F4 group in whom the PI value was 0 in many patients. Thus, further study is needed in this regard on a larger number of patients, including those with claudication.

In analysis 2, the CLI cutoff values were set for each PI index, and whether these could be used to predict severity in the F2 group were evaluated in analysis 3. The results showed that only the cutoff value of mean PI could classify the severe IC patient from the mild IC patients, thus, this cutoff value could be used to define and predict the progression of the disease. The sensitivity, specificity, positive predictive value, and negative predictive value at this point were 83.3%, 62.5%, 25%, and 96%, respectively, suggesting that patients with values at or above the cutoff value could be considered as having a high possibility of not developing severe symptoms and so, conservative therapy could be continued safely. Although the positive predictive value was low, careful follow-up should be necessary in conjunction with clinical symptoms when the PI value is lower than cutoff value, judging from the results of analysis 1. Moreover, there is a possibility that measurements were performed in a hypersympathetic state; thus, there is a need to consider repeating the measurements several times.

The limitation of the present study was that there were only 6 limbs included in the severe IC group from the F2 group. However, the cutoff value could be set even in this evaluation of a small number of patients, and the severity could be predicted to some extent; this may support the findings that PI is a sensitive index. There is a need to prospectively investigate whether the CLI cutoff value of 0.27 at the mean PI value is universally correct as a cutoff value and whether PI can be used to predict the development of CLI in patients with mild symptoms in the future.

## Conclusion

Ischemic screening for PAD can be performed easily and non-invasively using PI. We found that the possibility of progression to CLI is low if the mean PI is ≥0.27.

## Addendum

This study was reviewed and approved by the Institutional Review Board of Kansai Medical University Medical Center (Approval No.: T28-19) and was supported by the Japan Society for the Promotion of Science (JSPS) KAKENHI Grant Number JP19K18195. This study was conducted after written informed consent to participate in the study was obtained from the target patients after providing them with an explanation regarding the study.

## Notes

This is a translation of Jpn J Vasc Surg 2020; 29: 103–108.

## References

[1] Komai H, Honda K, Juri M, et al. Bypass operation for peripheral arterial disease associated with rheumatic disease. J Jpn Coll Angiol 2005; 45: 533-9.

[2] Komai H, Kawago M, Juri M. Prevalence and outcomes of thrombophilia defects with peripheral arterial disease. J Jpn Coll Angiol 2006; 46: 405-10.

[3] Komai H, Juri M, Shibata M, et al. Novel risk factors for graft occlusion after infrainguinal bypass operation for peripheral arterial disease. J Jpn Coll Angiol 2007; 47: 439-44.

[4] Komai H, Higami Y, Tanaka H, et al. Impaired flow-mediated endothelium-dependent and endothelium-independent vasodilation of the brachial artery in patients with atherosclerotic peripheral vascular disease. Angiology 2008; 59: 52-6. 18319222 10.1177/0003319707303442

[5] Komai H, Shibata R, Juri M, et al. Plasma adiponectin as predictive factor of survival after a bypass operation for peripheral arterial disease. J Vasc Surg 2009; 50: 95-9. 19563957 10.1016/j.jvs.2008.12.044

[6] Komai H, Shindo S, Sato M, et al. Reduced protein C activity might be associated with progression of peripheral arterial disease. Angiology 2015; 66: 584-7. 25115555 10.1177/0003319714544946

[7] Komai H, Miyama N, Sakashita H, et al. Plasma carnitine level in peripheral arterial disease. J Jpn Coll Angiol 2016; 56: 103-8.

[8] Ito K. New algorithm, Perfusion Index (PI), and Pleth Variability Index (PVI)—how to utilize them in general anesthesia management. JJSCA 2011; 31: 501-6.

[9] De Felice C, Latini G, Vacca P, et al. The pulse oximeter perfusion index as a predictor for high illness severity in neonates. Eur J Pediatr 2002; 161: 561-2. 12297906 10.1007/s00431-002-1042-5

[10] Ohmine S. Clinical Practice Guideline for Physical Therapy of Lower Limb Amputation. Physical Therapy Japan 2015; 42: 296-304. (in Japanese)

[11] Shirasu T, Hoshina K, Yamamoto S, et al. Poor prognosis in critical limb ischemia without pre-onset intermittent claudication. Circ J 2015; 79: 1618-23. 25925843 10.1253/circj.CJ-15-0017

[12] Dormandy J, Belcher G, Broos P, et al. Prospective study of 713 below-knee amputations for ischaemia and the effect of a prostacyclin analogue on healing. Br J Surg 1994; 81: 33-7. 7508804 10.1002/bjs.1800810110

[13] Azuma N, Uchida H, Kokubo T, et al. Factors influencing wound healing of critical ischemic foot after bypass surgery: is the angiosome important in selecting bypass target artery? Eur J Vasc Endovasc Surg 2012; 43: 322-8. 22237509 10.1016/j.ejvs.2011.12.001

[14] Goldman JM, Petterson MT, Kopotic RJ, et al. Masimo signal extraction pulse oximetry. J Clin Monit Comput 2000; 16: 475-83. 12580205 10.1023/a:1011493521730

[15] Yoshimuta T, Matsuo H. Medical treatment for peripheral artery disease. Kokyu To Junkan 2011; 59: 611-7.

[16] Abe T, Kimata N, Otsubo S, et al. Tousekikanjyano masyoudoumyakushikkannitaisuru ABI, SPP no yuuyousei. Nihon Toseki Igakkai Zasshi 2016; 49: 669-76. (in Japanese)

