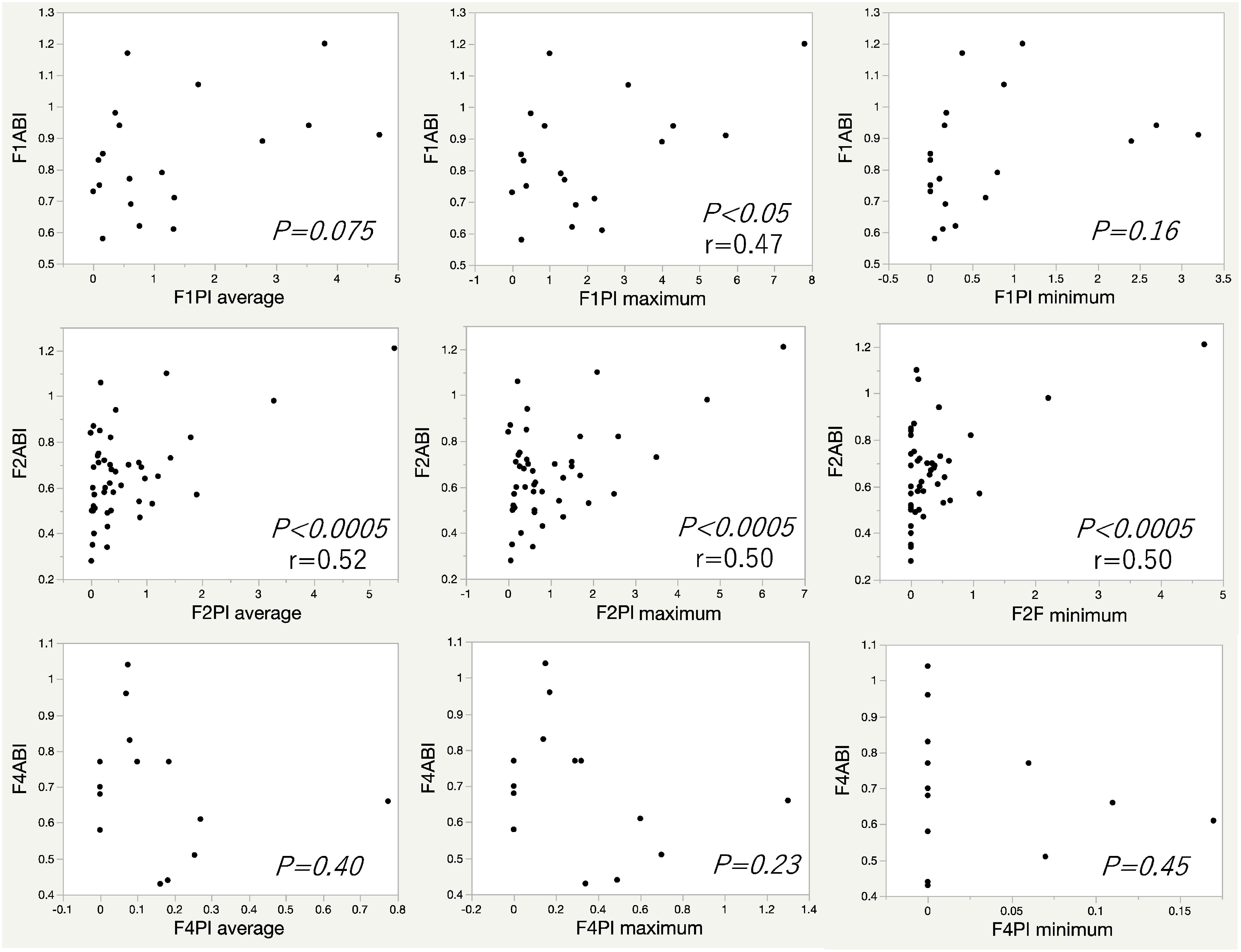# ERRATUM

**DOI:** 10.3400/avd.err.22-01000

**Published:** 2022-03-25

Vol. 14 (2021) No. 4 pp. 328–333

Evaluation of Perfusion Index as a Screening Tool for Developing Critical Limb Ischemia

Nobuko Yamamoto, Hideki Sakashita, Noriyuki Miyama, Kanako Takai, Hiroyoshi Komai

In the above secondary publication article, an error was found after the publication. The erratum of the original Japanese article was published in Japanese Journal of Vascular Surgery Vol. 31 (2022) No. 2. The corrected version of **Fig. 5** is given below.

p. 331, **Fig. 5** Correlation analysis between ABI and each PI value in each group. There are moderate correlations between ABI and PI maximum value in F1 group and all PI value in F2 group.

Incorrect:

**Figure:**
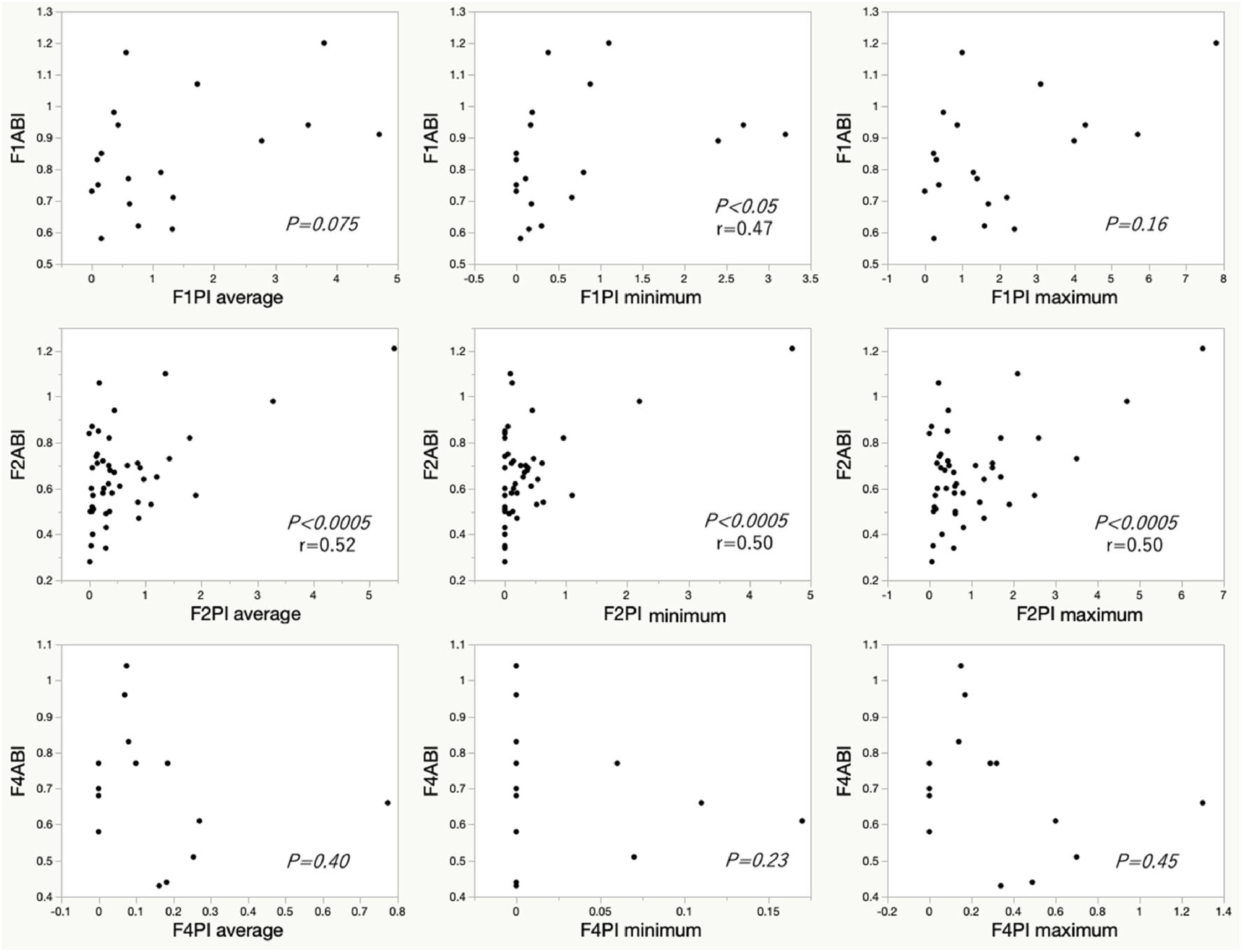


Correct:

**Figure:**