# ERRATUM

**DOI:** 10.3400/avd.err.22-01001

**Published:** 2022-09-25

Vol. 14 (2021) No. 4 pp. 328–333

Evaluation of Perfusion Index as a Screening Tool for Developing Critical Limb Ischemia

Nobuko Yamamoto, Hideki Sakashita, Noriyuki Miyama, Kanako Takai, Hiroyoshi Komai

In the above secondary publication article, errors were found after the publication. The erratum of the original Japanese article was published in Japanese Journal of Vascular Surgery Vol. 31 (2022) No. 3. The same corrections are given below.

p. 328, Abstract, ll. 15–18

Incorrect:

In 65 asymptomatic PAD patients and claudication, significantly more patients with a PI value greater than the cut-off value developed CLI than those with a PI lower than the cut-off.

Correct:

In 65 asymptomatic PAD patients and claudication, significantly more patients with a PI value greater than the cut-off value did not develop CLI than those with a PI lower than the cut-off.

p.328, Right, Patients and Methods, ll. 1–4

Incorrect:

Seventy-nine limbs of 70 patients (56 limbs in males, 23 limbs in females) with PAD (ankle-brachial index [ABI] <0.9) who presented to our department between March 2015 and August 2016 were included in the study.

Correct:

Seventy-nine limbs of 70 patients (56 limbs in males, 23 limbs in females) with PAD who presented to our department between March 2015 and August 2016 were included in the study.

